# Distribution of Marine Lipophilic Toxins in Shellfish Products Collected from the Chinese Market

**DOI:** 10.3390/md13074281

**Published:** 2015-07-14

**Authors:** Haiyan Wu, Jianhua Yao, Mengmeng Guo, Zhijun Tan, Deqing Zhou, Yuxiu Zhai

**Affiliations:** 1 Carbon-sink Fisheries Laboratory, Yellow Sea Fisheries Research Institute, Chinese Academy of Fishery Sciences, Qingdao 266071, China; E-Mails: wuhy@ysfri.ac.cn (H.W.); yaojianhua@cti-cert.com (J.Y.); guomm@ysfri.ac.cn (M.G.); zhoudq@ysfri.ac.cn (D.Z.); zhaiyx@ysfri.ac.cn (Y.Z.); 2 Key Laboratory of Testing and Evaluation for Aquatic Product Safety and Quality, Ministry of Agriculture, Qingdao 266071, China; 3 National Center for Quality Supervision and Test of Aquatic Products, Qingdao 266071, China

**Keywords:** ESI-LC-MS/MS, identification, distribution, lipophilic toxins, shellfish

## Abstract

To investigate the prevalence of lipophilic marine biotoxins in shellfish from the Chinese market, we used hydrophilic interaction liquid chromatography-tandem mass spectrometry (LC-MS/MS) to measure levels of okadaic acid (OA), azaspiracid (AZA1), pectenotoxin (PTX2), gymnodimine (GYM), and spirolide (SPX1). We collected and analyzed 291 shellfish samples from main production sites along a wide latitudinal transect along the Chinese coastline from December 2008 to December 2009. Results revealed a patchy distribution of the five toxins and highlighted the specific geographical distribution and seasonal and species variation of the putative toxigenic organisms. All five lipophilic marine biotoxins were found in shellfish samples. The highest concentrations of OA, AZA1, PTX2, GYM, and SPX1 were 37.3, 5.90, 16.4, 14.4, and 8.97 μg/kg, respectively. These values were much lower than the legislation limits for lipophilic shellfish toxins. However, the value might be significantly underestimated for the limited detection toxins. Also, these toxins were found in most coastal areas of China and were present in almost all seasons of the year. Thus, these five toxins represent a potential threat to human health. Consequently, studies should be conducted and measures should be taken to ensure the safety of the harvested product.

## 1. Introduction

Harmful algal species produce toxins that can accumulate in shellfish, leading to ecological perturbations, economic losses, threats to public health, and concerns about quality of shellfish products. In recent years, lipophilic marine biotoxins have become a worldwide problem. However, the tolerance limits and analytical methods used to ensure compliance to such limits differ significantly among countries. These differences should be eliminated in order to develop consistent rules for protection of public health and for greater harmonization of international trade. The regulatory structure in the European Union (EU) includes a series of regulations for the control of lipophilic toxins. Regulation (EC) N° 853/2004 sets the maximum levels for lipophilic toxins in bivalve molluscs being placed on the market for human consumption. Commission regulation (EU) No 15/2011 [1], which amends regulation (EC) No 2074/2005, recognizes liquid chromatography-tandem mass spectrometry (LC-MS/MS) testing methods for the detection of lipophilic toxins in live bivalve mollusks, and this technique is used for routine detection of toxins.

Currently, China is the main bivalve-culturing country. However, due to the lack of shellfish safety control measures, many countries do not allow the import of Chinese-cultured bivalves, and almost all the bivalves produced are sold in the Chinese market. A lack of monitoring and management programs for shellfish toxins leave consumers easily exposed to contaminated shellfish. In addition, traditional Chinese methods of processing shellfish, such as cooking, steaming, and autoclaving, increase the concentration of lipophilic marine biotoxins (okadaic acid (OA)-, azaspiracid (AZA)-, pectenotoxin (PTX)-, and yessotoxin (YTX)-group toxins) approximately two-fold due to water loss [2]. Occasional but serious poisoning events, which led to more than 200 people suffering illness, occurred in the cities of Ningbo and Ningde near the East China Sea in 2011 [3,4].

In recent years, the presence of many lipophilic marine biotoxins and the phytoplankton that produce them have been recorded frequently along the coast of China [5]. Members of the OA group are considered to be the most widely distributed toxins, as they have been found in mussels, oyster, clams and scallops [3,6,7]. Other lipophilic shellfish toxin groups, such as PTX, YTX, and gymnodimines (GYM), have been found in shellfish [5,6] or seawater [8]. In addition, toxic microalgae that produce lipophilic shellfish toxins have been found, such as *Dinophysis* and *Prorocentrum lima* (likely producers of OA, DTX and PTX), *Gymnodinium* (GYM producer) [9,10], *Azadinium poporum* (AZA producer) [11,12,13,14]. The apparent range expansion of toxic phytoplankton and their associated toxins along the coast of China poses a persistent threat of exposure to lipophilic shellfish toxins for consumers.

In this study, the prevalence of five different lipophilic shellfish toxins (OA, AZA1, PTX2, GYM, and SPX1) along the Chinese coast was studied. Shellfish samples (clams/cockles, oysters, scallops and mussels) were collected monthly from coastal city markets from December 2008 to December 2009. The goals of this study were to provide a basic understanding of the current contamination situation of shellfish products in China and to provide data for the establishment of a monitoring program and market access system.

## 2. Materials and Methods

### 2.1. Reagents and Materials

Water was deionized and passed through a Milli-Q water purification system (Millipore, Billerica, MA, USA). Formic acid (>98%), ammonium acetate (>97%), and acetonitrile and methanol (absolute, hypergrade) were purchased from Merck (Darmstadt, Germany). Lipophilic toxin standards for OA (CRM-OA-b, 30.0 µmol/L), PTX (CRM-PTX2, 10.0 µmol/L), AZA (CRM-AZA1, 1.47 µmol/L), GYM (CRM-GYM, 10.0 µmol/L), and SPX (CRM-SPX1, 10.2 µmol/L), were purchased from the National Research Council Canada (Marine Analytical Chemistry Standards Program, Halifax, NS, Canada).

### 2.2. Collection and Preparation of Commercially Available Shellfish Samples

**Figure 1:**
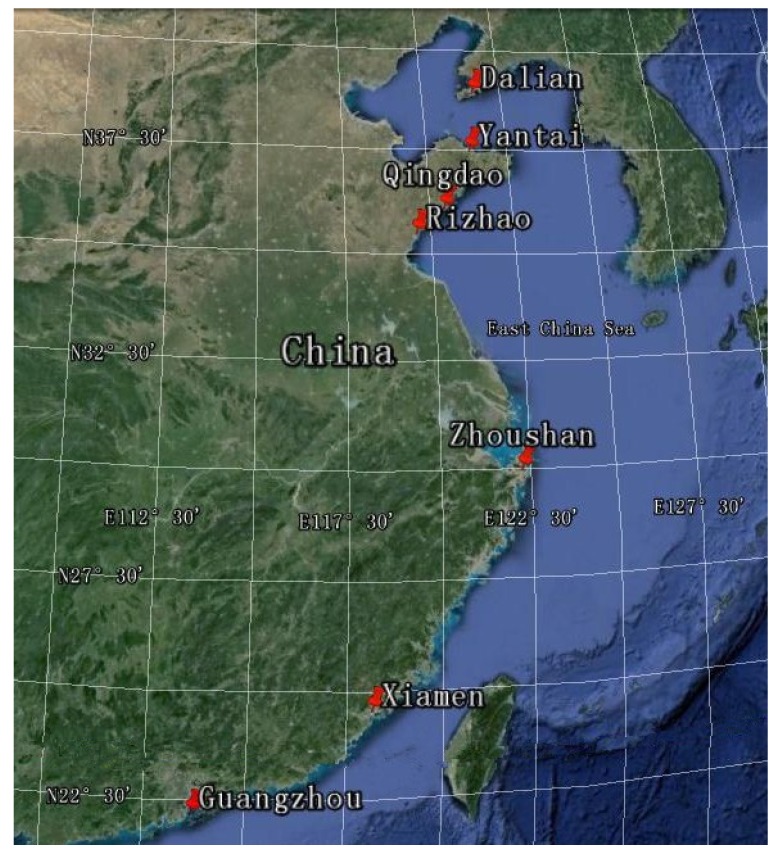
Sample collection cities along the Chinese coastline.

Shellfish samples were collected monthly from December 2008 to November 2009 from the main seafood markets in the following cities along the Chinese coastline: Guangzhou (GZ), Xiamen (XM), Zhoushan (ZS), Rizhao (RZ), Qingdao (QD), Yantai (YT), and Dalian (DL) (Figure 1). Shellfish samples consisted of the main cultured categories: clams (*Ruditapes philippinarum*, *Atrina pectinate*, *Mercenaria mercenaria*), mussels (*Perna viridis*, *Mytilus galloprovincialis*), scallops (*Chlamys farreri*, *Arca granosa*), and oysters (*Crassostrea gigas*).

All samples were sorted by location and specimens, washed with clean water, kept in portable incubators at 4 °C, and transported to the lab within 48 h. The muscle and digestive gland of each specimen were removed, labeled, and stored immediately in a freezer at −18 °C for later analysis. Samples were processed as described previously [15]. Briefly, 2.00 ± 0.02 g of tissue were extracted with 3 mL of methanol three times. The supernatants were combined and evaporated at 40 °C under nitrogen to less than 1 mL and diluted with 3 mL of water for purification. All 4 mL of diluted extract were loaded on a Strata-X cartridge (3 mL, 60 mg, Phenomenex, Torrance, CA, USA).The cartridge was washed with 1 mL of 20% v/v methanol and eluted with 1 mL of methanol containing 0.3% v/v ammonium hydroxide for liquid chromatography-tandem mass spectrometry (LC-MS/MS) analysis.

### 2.3. LC-MS/MS Analyses of Lipophilic Toxins

The LC-MS/MS system consisted of Thermo electrospray ionization-mass spectrometry (TSQ Quantum Access™, Thermo Electron Corporation, Madison, WI, USA) and a Finnigan HPLC system that included a quaternary pump and a thermostatted autosampler to maintain the sample vials at 4 °C. The Atlantis™ dC18 analytical column (150 mm × 4.6 mm, i.d., 5.0 μm, Waters, Milford, MA, USA) was maintained at 35 °C. Mobile phase A was acetonitrile/water (95/5, v/v) and B was water, and both contained 2 mM ammonium formate and 50 mM formic acid. A gradient was run at a flow rate of 0.2 mL/min starting at 40% A, which was increased linearly to 96% A in 2.1 min, kept constant for 4 min, then returned to 40% in 2 min. The total run time for the analysis was 9 min. For the first and last minute of the chromatographic run, the LC eluent was diverted to waste.

The LC-MS/MS experiment was carried out using a TSQ mass spectrometer equipped with an electrospray ionization (ESI) source, and it was operated in positive polarity for GYM, SPX1, PTX2, AZA1 and negative polarity for OA measurements. The voltage on the ESI needle was set at 4 kV, producing a spray current of approximately 80 mA. The capillary voltage was set at 10 V, and the temperature of the heated capillary was 350 °C. The sheath gas pressure used was 25 psi and the auxiliary gas was 10 psi. The microscan width (m/z) was set at 0.01 and the scan time was 0.5 s. These parameters were previously optimized using toxin standards. The mass spectrometer was operated in selected reaction monitoring mode, analyzing the two most intense product ions per compound (one for quantitation and the other for confirmation). For ESI negative polarity, the transitions selected were: OA, 803.0 > 255.0 (49ev)/208.8 (47 ev). For ESI positive polarity, the transitions selected were: GYM, 508.3 > 490.6 (23ev)/174.2 (38 ev); SPX1, 692.5 > 674.0 (23ev)/444.5 (34 ev); PTX, 876.0 > 823.2 (21 ev)/212.8 (36 ev); AZA1, 842.5 > 824.5 (30 ev)/672.4 (38 ev). The most abundant ions in the fragment spectra were used for quantitation: 225.0 (OA), 490.6 (GYM), 674.0 (SPX1), 823.2 (PTX), and 824.5 (AZA1).

## 3. Results and Discussion

### 3.1. Evaluation of the Detection Method for Five Lipophilic Toxins by LC-MS/MS

**Figure 2:**
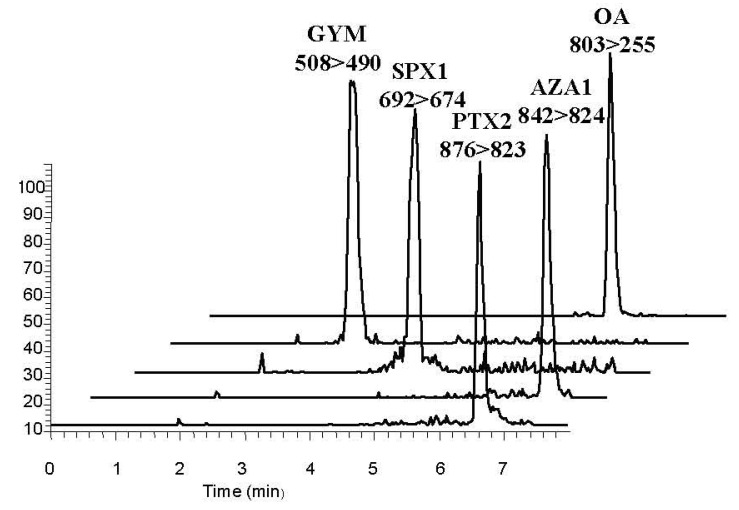
Chromatograms of a blank shellfish muscle extract spiked with GYM, SPX1, OA, PTX2, and AZA1 (concentrations are 0.63, 1.77, 20.0, 4.29 and 0.48 μg/kg, respectively).

Parameters such as recovery, standard deviation (SD), and relative standard deviation (RSD) (Table 1) were investigated to evaluate the sensitivity of the LC-MS/MS method using a blank spiked sample. The peaks obtained were symmetrical and fully separated within 7 min (Figure 2). To determine the limit of detection for each toxin, a signal-to-noise ratio of 3 was extrapolated from the lowest abundant product ion of the toxin present in the lowest spiked mussel extract. The limits of detection for OA, AZA1, PTX2, GYM, and SPX1 were 2.00, 0.04, 0.32, 0.10, and 0.21 μg/kg, respectively. The calibration curves between areas and the concentration of the five lipophilic marine toxins were linear and had correlation coefficients >0.99. The average recoveries from spiked scallop muscle at the three concentrations tested ranged from 78.6% to 94.4% with RSDs from 6.80% to 14.9%. Therefore, the precision of the method meets the distribution investigation needs for these five marine lipophilic toxins in shellfish.

**Table 1:** Recoveries and precisions of the five lipophilic marine toxins in blank samples (*n* = 6).

Toxins	Fortification Level (μg/kg)	Repeatability Mean Concentrations (µg/kg) ± SD (µg/kg)	Mean Recovery (%)	RSD (%)
GYM	0.25	0.21 ± 0.02	85.8	8.40
	0.63	0.57 ± 0.07	91.0	12.4
	1.26	1.13 ± 0.11	89.7	10.0
SPX1	0.88	0.70 ± 0.05	79.3	7.70
	1.77	1.66 ± 0.25	93.8	14.9
	3.53	3.24 ± 0.04	91.8	10.8
OA	10.0	7.86 ± 0.16	78.6	10.2
	20.0	17.8 ± 2.08	88.9	11.7
	30.0	28.3 ± 3.17	94.4	11.2
PTX2	2.15	1.74 ± 1.27	81.1	7.30
	4.29	3.83 ± 0.41	89.2	10.8
	6.44	5.65 ± 0.38	87.7	6.80
AZA1	0.24	0.21 ± 0.03	88.7	12.7
	0.48	0.43 ± 0.04	89.3	10.1
	0.72	0.65 ± 0.09	90.1	13.3

### 3.2. Spatial Variation of Lipophilic Toxins in Shellfish Products

In this investigation, bivalves were collected from seven Chinese cities near important areas of shellfish culture distributed along the coastline from south to north. OA, AZA1, PTX2, GYM, and SPX1 were detected in 25.8%, 2.75%, 10.6%, 15.1%, and 2.43% of the 291 samples analyzed, respectively. Although shellfish products in every city were contaminated with at least some of these lipophilic marine biotoxins, the toxin concentration and composition varied widely among the various cities.

**Figure 3:**
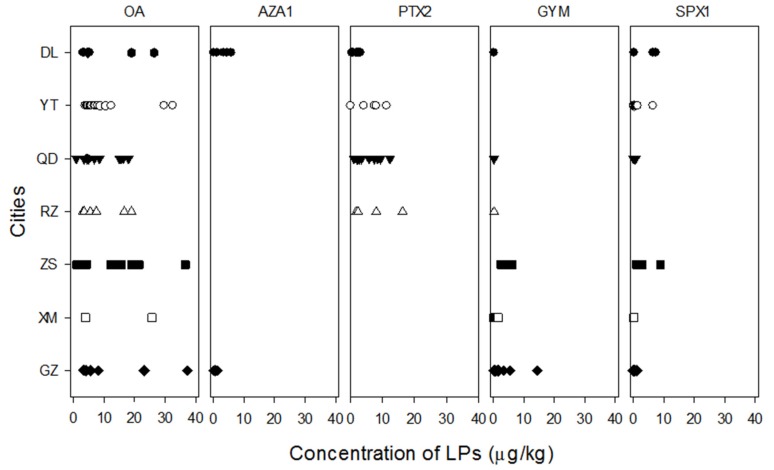
Variation of lipophilic marine biotoxins in shellfish products collected from seven cities along the coast of China from December 2008 to November 2009.

Overall, three distinct patterns of distribution were observed (Figure 3). First, the two cities located in the southernmost (GZ) and northernmost (DL) locales showed a similar trend of lipophilic marine biotoxin contamination. Most of the lipophilic marine biotoxins, except for PTX2 in GZ, were present in a large percentage of specimens from these two cities. GYM was present in ~50% of samples from GZ, and the maximum level (14.4 μg/kg) was found here. The maximum concentration of OA (37.3 μg/kg) also occurred in this city. AZA1 was detected in shellfish at a maximum concentration as 5.90 μg/kg from DL. The presence of AZA and its microalga producer *A. poporum* along the coast of China [11] might mean that this toxin is now a new risk to consumers. However, the prevalence and concentration of AZA1 were much lower than those of the other lipophilic shellfish toxins.

The second pattern involved the shellfish products from XM and ZS, both of which are located along the coast of the East China Sea. In both cities, specimens were contaminated with OA, GYM, and SPX1. However, the situation in ZS was more serious than that in XM, especially for OA from 21.4 to 36.6 μg/kg. Finally, specimens from RZ, QD, and YT, which lie along the coast of the Yellow Sea, showed relatively low concentrations of the lipophilic shellfish toxins. The exception was PTX2, which was present at 16.4 μg/kg in QD.

In China, OA is recognized as the main cause of diarrheic shellfish poisoning (DSP) [3,4,8,13]. Among the five toxins tested in this study, OA was the only one found in all locations, with the highest prevalence (37.8%) occurring in QD. GYM and SPX1 were also distributed along most parts of the coastline. The highest prevalence of GYM was 71.9% in GZ, and that of SPX1 was 52.4% in RZ. GYM was mostly observed in coastal water of the South China Sea, whereas PTX2 was distributed mainly in northern China along the coast of the Bohai Sea [16] and the East China Sea [13] , and the concentrations were relatively low (0.11–9.42 µg/kg). AZA1 was only detected in GZ and DL, which are located in the south and north of China, respectively.

Although OA, PTX2, AZA1, GYM, and SPX1 were detected in the shellfish products analyzed in this study, they were present at relatively low concentrations that were under the limit set by the EU to ensure that shellfish products are safe for human consumption.

### 3.3. Seasonal Variation of Lipophilic Toxins in Shellfish Products

Toxin-forming organisms are known to occur periodically, and the toxins are prone to accumulation in shellfish. Seasonal variations in the presence and levels of microalgae toxins in the waters, phytoplankton, and shellfish are strongly related. Many nations with monitoring programs use cell counts of various toxin-producing algae as an indicator to increase monitoring or initiate precautionary harvesting restrictions (e.g., when cell concentrations of lipophilic shellfish toxin-producing organisms exceed 100–1000 cells/L [17]). Nevertheless, toxin production by algae varies with time and the species present, and the number of cells alone cannot be used as an indicator for the presence of toxins.

Toxin absorption and elimination rates as well as temperature and pH of the seawater are important factors that can affect the growth of different microalgae and the ability of shellfish to accumulate toxins [18]. In addition, size of bivalves has a significant effect on their ingestion rate. As shown in Table 2, three out of the five toxins were present in spring when bivalves are relatively large in size; in particular, the concentration of OA reached 37.3 µg/kg in spring. In contrast, shellfish are relatively small in autumn, and the prevalence and concentration of lipophilic shellfish toxins were relatively low during this season. The large differences in toxin prevalence and concentration in summer might be due to the diverse growth habits of different toxin-producing algae.

PTXs always appear together with toxins from the OA group because they are produced by the same dinoflagellate genus, *Dinophysis* [19]. PTX and OA both showed high prevalence and concentration in winter and spring. In the third and fourth quarters of the year, PTX2 was present in <6% of the samples, probably because the growth of PTX2-producing algae was inhibited. This likely also explains why PTX2 was not detected in the southern coastal cities. GYM and SPX1 were detected in almost all seasons in different shellfish samples, although the highest levels of GYM occurred in winter. This result matches the data that detected from samples in May 2012 from the East China Sea [13].

Due to the large latitudinal span of the coastal zone in China, the occurrence of red tides differs greatly over both space and time. Although the lag between the seasonal conditions and outbreaks of lipophilic shellfish toxin poisoning is compatible with the obligate mixotrophic nutritional mode of the *Dynophysis acuminate* complex [20], predicting when and where blooms will occur remains difficult. To ensure the safety of shellfish products in China, an annual monitoring program should be in effect throughout the entire year, with more intense sampling during shellfish harvest periods.

**Table 2:** Seasonal variability and contents of lipophilic toxins in shellfish samples from China (µg/kg).

Seasons	OA	AZA1	PTX2	GYM	SPX1
**Winter** (2008.12–2009.2)					
Content span(µg/kg)	2.87–32.5	-	0.53–**16.4** ^a^	0.28–**14.4** ^a^	0.23–7.45
Median(µg/kg)	4.89	-	3.84	0.78	0.67
% of samples quantified	29.8	-	**14.3** ^b^	11.9	14.3
Sample size	84.0	84.0	84.0	84.0	84.0
**Spring** (2009.3–2009.5)					
Content span(µg/kg)	2.00–**37.3** ^a^	0.18–1.37	1.04–11.4	0.10-8.11	0.21–1.50
Median(µg/kg)	5.58	0.88	2.90	0.73	0.58
% of samples quantified	**36.8** ^b^	**4.60** ^b^	12.6	14.9	**18.4** ^b^
Sample size	87.0	87.0	87.0	87.0	87.0
**Summer** (2009.6–2009.8)					
Content span(µg/kg)	3.12–19.0	0.12–4.56	1.1–12.4	0.13-6.26	0.25–**8.97** ^a^
Median(µg/kg)	5.07	3.41	3.21	1.22	0.57
% of samples quantified	21.9	4.11	6.85	**24.6** ^b^	15.1
Sample size	73.0	73.0	73.0	73.0	73.0
**Autumn** (2009.9–2009.11)					
Content span(µg/kg)	3.52–7.41	**5.90** ^a^	0.64–2.96	0.14-2.70	6.58
Median(µg/kg)	4.58		2.10	1.42	
% of samples quantified	6.38	2.13	6.38	4.26	2.13
Sample size	47.0	47.0	47.0	47.0	47.0

^a^ Means the maximum concentration of each kind of lipophilic shellfish toxins detected during this investigation; ^b^ means the highest % of samples quantified of each kind of lipophilic shellfish toxins in this investigation.

### 3.4. Variation of Lipophilic Shellfish Toxins in Different Shellfish Species and Tissue Distribution

**Figure 4:**
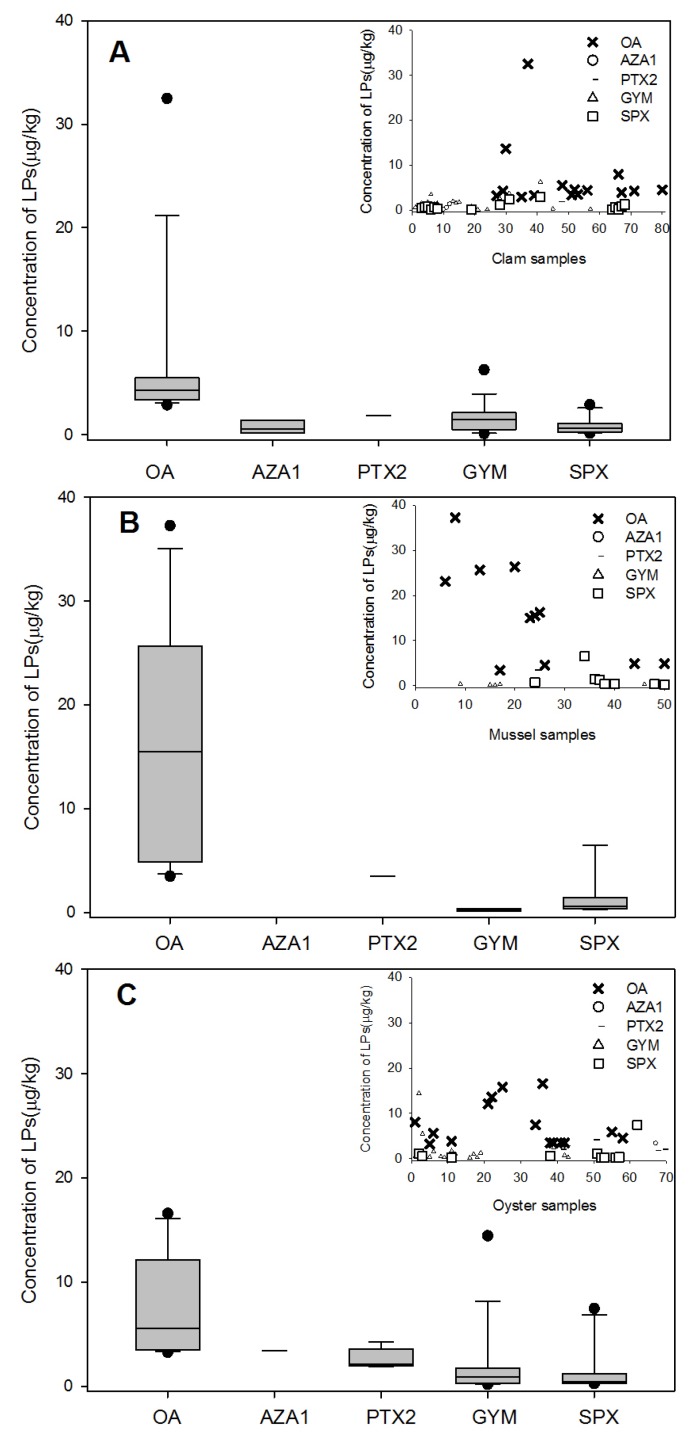

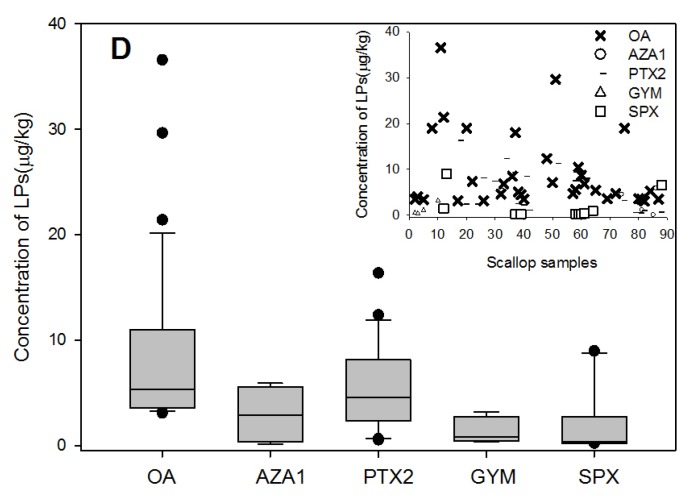
Contamination of lipophilic shellfish toxins in difference shellfish species: (**A**) 82 clam samples collected from markets in all seven cities; (**B**) 51 mussel samples collected from five cities (excluding Zhoushan and Qingdao); (**C**) 69 oyster samples from markets in all seven cities; (**D**) 89 scallop samples collected from six cities (excluding Zhoushan). The bottom and top of the box are the first and third quartiles, the band inside the box is the second quartile (the median), the ends of the whiskers represents one standard deviation above and below the mean of the data, and the dots are mild outliers.

The feeding behaviors of different bivalves, particularly their maximum filtration rates, affect their ability to accumulate and bio-transform toxins in different tissues. DSP toxins have been recognized as a serious threat to public health and have attracted significant public attention in China. OA was the primary DSP toxin detected in all four types of shellfish tested in our study, and it was present at relatively high levels. Previous studies reported that mussels had the greatest accumulation ability [21]. Concentrations of toxins in Pacific oysters and Manila clams were often at least half those measured in blue mussels at the same site [22], and the DSP toxin content was about 10 times higher in mussels than in oysters collected from the same region [23]. The first recorded outbreak in China was attributed to the consumption of mussels contaminated by DSP toxins (OA and DTX-1), whereas consumption of other seafood items was not associated with illness [4]. The data obtained in our investigation are consistent with these findings, as the OA level in mussel samples was highest (Figure 4B), followed by that in scallop samples.

Toxin profiles differ not only among different geographic regions but also among populations within the same geographic origin [12]. With the exception of OA, the accumulation of the other toxins (AZA1, PTX2, GYM, SPX) was relatively high in scallop samples compared to the other species (Figure 4D). For the AZA group, AZA1 was the predominant toxin in mussels and AZA2 was the predominant toxin in scallops [24]. This finding illustrates that mussels and scallops have different feeding selectivity for toxin-producing algae. Similarly, a remarkable difference in toxin levels and profiles was observed between mussels and oysters from the same station during the survey period.

All five lipophilic toxins in shellfish were concentrated mainly in the digestive gland, which is consistent with results of the James study [25]. Although GYM was detected both in the digestive gland and the edible parts, the concentration in the digestive gland was about 20 times higher than that in the edible parts. This result suggests that the toxin is concentrated almost exclusively in the digestive gland, which is a behavior very similar to that of other lipophilic toxins. However, as the remaining flesh represents 85% of total mussel flesh [26], it still has a considerable sanitary impact, meaning the whole shellfish should be analyzed for official control purposes. It has been shown that toxin concentrations in king scallops were lower in samples in which the viscera had been removed than in whole organism samples [27]. Therefore, to avoid the ingestion of lipophilic toxins, the digestive parts should be removed prior to consumption.

In addition to the effects of feeding rate, particle selection, and behavioral responses on the accumulation of biotoxins by shellfish, the rates of detoxification and biotransformation should be considered when evaluating the differential toxin retention of different species. Balancing between accumulation and depuration of toxins in bivalves after they have consumed lipophilic shellfish-toxin-producing organisms could last for a long time [14].

### 3.5. Potential Risk of Lipophilic Shellfish Toxins in Shellfish Products

The main threat to shellfish raft culture is the recurrent appearance of harmful algal bloom species that produce lipophilic toxins that can be transferred through the food web. Ironically, because filter-feeding shellfish do not need dense blooms of toxic algae to eventually accumulate amounts of toxin deadly to humans, many of the most serious algal-related human health hazards are not necessarily associated with dense obvious blooms. In addition, most official monitoring programs analyze raw shellfish tissue to determine the content of lipophilic toxins. However, most shellfish are eaten cooked or steamed, so heat-treated tissue seems to be the most relevant form to analyze. Moreover, heat treatment can lead to a two-fold concentration of toxins due to water loss. These issues should be recognized in relation to future risk assessments to establish and control permissible toxin levels.

In our study, most of the shellfish specimens were contaminated with a relatively low level of lipophilic shellfish toxins. The maximum levels of OA, AZA1, PTX2, GYM, and SPX1 were 37.3, 5.90, 16.4, 14.4, and 8.97 μg/kg respectively, which are much lower than the regulatory limits in shellfish flesh (Table 3). However, as the standards were not available to us, some important toxins such as DTX1 and AZA2, were not screened. The content of marine lipophilic toxins might be significantly underestimated, and potential risk to local consumers might still exist. For example, DSP was present at levels above the regulatory limit for human consumption (20 mg/100 g soft tissue) in 1999 [7] and 2011 [3,4]. In recent years, the traditional mouse detection methods have been gradually replaced by LC-MS/MS or qualitative-only methods in China. Positive DSP samples had been specific to toxins, and DTX1 also showed a high positive rate and content [5,13], which should have been taken into consideration. Prevalence of this situation likely is higher, but data are scarce and monitoring programs and a market access system are absent in China.

**Table 3:** Limits for lipophilic shellfish toxins.

Scientific Opinion and Regulation	Toxin(s)	product	Tolerance Level	Reference Method
National shellfish sanitation program[28]	DSP and AZP	Shellfish	0.16 mg OA eq/kg 0.16 mg AZA-1 eq/kg	Mouse bioassay LC-MS
European Food Safety Authority [2]	OA,AZA and PTX	Shellfish	30 μg AZA eq/kg 45 μg OA eq/kg 120 μg PTX eq/kg	Mouse bioassay LC-MS
Codex Alimentarius Commission [29]	OA, DTX and PTX YTX		0.16 mg OA eq/kg 1 mgYTX1eq/kg	Mouse bioassay LC-MS
European Union [1,30]	OA,DTXs, and PTXs together YTXs AZAs	Bivalve molluscs	0.16 mg OA eq/kg 1 mg YTX eq/kg 0.16 mg AZA eq/kg	LC-MS/MS

PTX was recorded previously in farmed shellfish [6] and seawater [31]. In this investigation, PTX was detected in both scallops and oysters. Considering the widespread distribution of *Dinophysis* [8,13], the producer of PTX, along the Chinese coast, PTX may prove to be a major health risk to consumers in China. AZA was only found in *Atrina pectinate* from GZ and scallops from DL, which are located in the south and north of China. The maximum concentrations of AZA1 (5.90 μg/kg) and PTX2 (16.4 μg/kg) were detected in the same scallop. Due to the large number of AZA-analogues that had been observed in shellfish as metabolites, the total AZAs content might be significantly underestimated in this study with the detection of AZA1 only. However, the microalga that produces AZAs (*A. poporum*) is present along the coast of China and exhibits highly variable toxin profiles [12]. Furthermore, Chinese traditional cooking methods might increase the concentration of AZAs approximately two-fold due to water loss, meaning this concentration would be above the recommended regulatory limit of 30 μg AZA1 equivalents/kg shellfish meat by EFSA [32]. Therefore, AZAs might become a new problem and cause significant economic losses to the shellfish industry and pose a potential threat to consumers in China. Further data are needed for ongoing assessment of the presence and variability of the AZAs toxin.

## 4. Conclusions

An LC-MS/MS method for simultaneous detection of GYM, SPX1, OA, PTX2, and AZA1 was used to determine the presence and distribution of five marine lipophilic toxins in shellfish along the coast of China. All five toxins were found in the shellfish digestive gland. Although the highest concentrations of these toxins were much lower than the permitted limits established by the EU, lipophilic toxins might be significantly underestimated due to the restricted toxin variety detected. Also, these toxins were present in most coastal areas of China at almost all times of the year, which shows that they are a major potential threat to human health. Consequently, risk assessment and monitoring of shellfish toxicity and its causative organisms should be conducted for both current and developing shellfish harvesting beds. More data on the timing and intensity of algal blooms and the subsequent toxin accumulation in shellfish can be used to develop a thorough monitoring strategy to track the occurrence of lipophilic shellfish toxins and to assess risk in order to ensure continued development of China’s shellfish production program.

## References

[1] European Union Reference Laboratory for Marine Biotoxins EU-Harmonised Standard Operating Procedure for Determination of Lipophilic Marine Biotoxins in Molluscs by LC-MS/MS. http://aesan.msssi.gob.es/CRLMB/docs/docs/metodos_analiticos_de_desarrollo/EU-Harmonised-SOP-LIPO-LCMSMS_Version5.pdf.

[2] European Food Safety Authority (2009). Scientific Opinion: Marine Biotoxins in Shellfish—Summary on Regulated Marine Biotoxins Scientific Opinion of the Panel on Contaminants in the Food Chain (Question No EFSA-Q-2009-00685). EFSA J..

[3] Li A., Ma J., Cao J., McCarron P. (2012). Toxins in mussels (*Mytilus galloprovincialis*) associated with diarrhetic shellfish poisoning episodes in China. Toxicon: Off. J. Int. Soc. Toxinol..

[4] Chen T., Xu X., Wei J., Chen J., Miu R., Huang L., Zhou X., Fu Y., Yan R., Wang Z. (2013). Food-borne disease outbreak of Diarrhetic shellfish poisoning due to toxic mussel consumption: The first recorded outbreak in China. PLoS ONE.

[5] Lin C., Liu Z., Tan C., Guo Y., Li L., Ren H., Li Y., Hu P., Gong S., Zhou Y. (2015). Contamination of commercially available seafood by key diarrhetic shellfish poisons along the coast of China. Environ. Sci. Pollut. Res. Int..

[6] Liu R., Liang Y., Wu X., Xu D., Liu Y., Liu L. (2011). First report on the detection of pectenotoxin groups in Chinese shellfish by LC-MS/MS. Toxicon: Off. J. Int. Soc. Toxinol..

[7] Zhou M., Li J., Luckas B., Yu R., Yan T., Hummert C., Kastrup S. (1999). A Recent Shellfish Toxin Investigation in China. Mar. Pollut. Bull..

[8] Li X., Li Z., Chen J., Shi Q., Zhang R., Wang S., Wang X. (2014). Detection, occurrence and monthly variations of typical lipophilic marine toxins associated with diarrhetic shellfish poisoning in the coastal seawater of Qingdao City, China. Chemosphere.

[9] Chen G., Wang G., Zhang C., Zhou B. (2008). Morphological and phylogenetic analysis of a Gymnodinium-like species from the Chinese Coast. Chin. Sci. Bull..

[10] Lu S., Hodgkiss I.J. (2004). Harmful algal bloom causative collected from Hong Kong waters. Hydrobiologia.

[11] Gu H., Luo Z., Krock B., Witt M., Tillmann U. (2013). Morphology, phylogeny and azaspiracid profile of *Azadinium poporum* (Dinophyceae) from the China Sea. Harmful Algae.

[12] Krock B., Tillmann U., Witt M., Gu H. (2014). Azaspiracid variability of *Azadinium poporum* (Dinophyceae) from the China Sea. Harmful Algae.

[13] Li A., Sun G., Qiu J., Fan L. (2015). Lipophilic shellfish toxins in *Dinophysis caudata* picked cells and in shellfish from the East China Sea. Environ. Sci. Pollut. Res. Int..

[14] Jiang T., Xu Y., Li Y., Qi Y., Jiang T., Wu F., Zhang F. (2014). *Dinophysis caudata* generated lipophilic shellfish toxins in bivalves from the Nanji Islands, East China Sea. Chin. J. Oceanol. Limnol..

[15] Wu H., Guo M., Tan Z., Cheng H., Li Z., Zhai Y. (2014). Liquid chromatography quadrupole linear ion trap mass spectrometry for multiclass screening and identification of lipophilic marine biotoxins in bivalve mollusks. J. Chromatogr. A.

[16] Liu R., Liang Y., Liu L., Fan D., Xu D., Sun Q. (2014). The lipophilic phycotoxins profile and distribution in bivalve shellfish of Chinese coasts by high performance liquid chromatography coupled with mass spectrometry. Ecol. Environ. Sci..

[17] Anderson D.M. (2001). Monitoring and Management Strategies for Harmful Algal Blooms in Coastal Waters.

[18] Webb J.L., Vandenbor J., Pirie B., Robinson S.M.C., Cross S.F., Jones S.R.M., Pearce C.M. (2013). Effects of temperature, diet, and bivalve size on the ingestion of sea lice (*Lepeophtheirus salmonis*) larvae by various filter-feeding shellfish. Aquaculture.

[19] Botana L.M. (2008). Seafood and Freshwater Toxins: Pharmacology, Physiology, and Detection,.

[20] Álvarez-Salgado X.A., Figueiras F.G., Fernández-Reiriz M.J., Labarta U., Peteiro L., Piedracoba S. (2011). Control of lipophilic shellfish poisoning outbreaks by seasonal upwelling and continental runoff. Harmful Algae.

[21] Viviani R. (1992). Eutrophication, marine biotoxins, human health. Sci. Total Environ..

[22] Trainer V.L., Moore L., Bill B.D., Adams N.G., Harrington N., Borchert J., Da S.D., Eberhart B.T. (2013). Diarrhetic shellfish toxins and other lipophilic toxins of human health concern in Washington State. Mar. Drugs.

[23] Lee K.J., Mok J.S., Song K.C., Yu H., Jung J.H., Kim J.H. (2011). Geographical and Annual Variation in Lipophilic Shellfish Toxins from Oysters and Mussels along the South Coast of Korea. J. Food Prot..

[24] Lopez-Rivera A., OʼCallaghan K., Moriarty M., OʼDriscoll D., Hamilton B., Lehane M., James K.J., Furey A. (2010). First evidence of azaspiracids (AZAs): A family of lipophilic polyether marine toxins in scallops (*Argopecten purpuratus*) and mussels (*Mytilus chilensis*) collected in two regions of Chile. Toxicon: Off. J. Int. Soc. Toxinol..

[25] James K.J., Lehane M., Moroney C., Fernandez-Puente P., Satake M., Yasumoto T., Furey A. (2002). Azaspiracid shellfish poisoning: Unusual toxin dynamics in shellfish and the increased risk of acute human intoxications. Food Addit. Contam..

[26] Furey A., Moroney C., Brana-Magdalena A., Saez M.J., Lehane M., James K.J. (2003). Geographical, temporal, and species variation of the polyether toxins, azaspiracids, in shellfish. Environ. Sci. Technol..

[27] Madigan T.L., Lee K.G., Padula D.J., McNabb P., Pointon A.M. (2006). Diarrhetic shellfish poisoning (DSP) toxins in South Australian shellfish. Harmful Algae.

[28] National Shellfish Sanitation Program: Guide for the Control of Molluscan Shellfish, 2013 Revision. http://www.fda.gov/downloads/Food/GuidanceRegulation/FederalStateFoodPrograms/UCM415522.pdf.

[29] (2012). Report of the Electronic Working Group on the Proposed Draft Performance Criteria for Screening Methods for Marine Biotoxins in the Standard for Raw and Bivalve Molluscs. ftp://ftp.fao.org/codex/meetings/ccffp/ccffp32/fp32_08e.pdf.

[30] Regulation (EC) No 853/2004 of the European Parliament and of the Council of 29 April 2004 Laying down Specific Rules for the Organisation of Official Controls on Products of Animal 
Origin Intended for Human Consumption. http://eur-lex.europa.eu/legal-content/EN/ALL/?uri=CELEX:32004R0854.

[31] Li Z., Mengmeng G., Shouguo Y., Qingyin W., Zhijun T. (2010). Investigation of Pectenotoxin Profiles in the Yellow Sea (China) Using a Passive Sampling Technique. Mar. Drugs.

[32] European Food Safety Authority Marine Biotoxins in Shellfish—Azaspiracid Group Scientific Opinion of the Panel on Contaminants in the Food chain.

